# Extreme Dewetting Resistance and Improved Visible Transmission of Ag Layers Using Sub-Nanometer Ti Capping Layers

**DOI:** 10.1021/acsomega.3c09774

**Published:** 2024-02-15

**Authors:** Amy L. Lynch, Christopher P. Murray, Evan Roy, Clive Downing, David McCloskey

**Affiliations:** † Technical University of Dublin, Grangegorman, Dublin D07 H6K8, Ireland; ‡ School of Physics, Trinity College, Dublin D02 PN40, Ireland

## Abstract

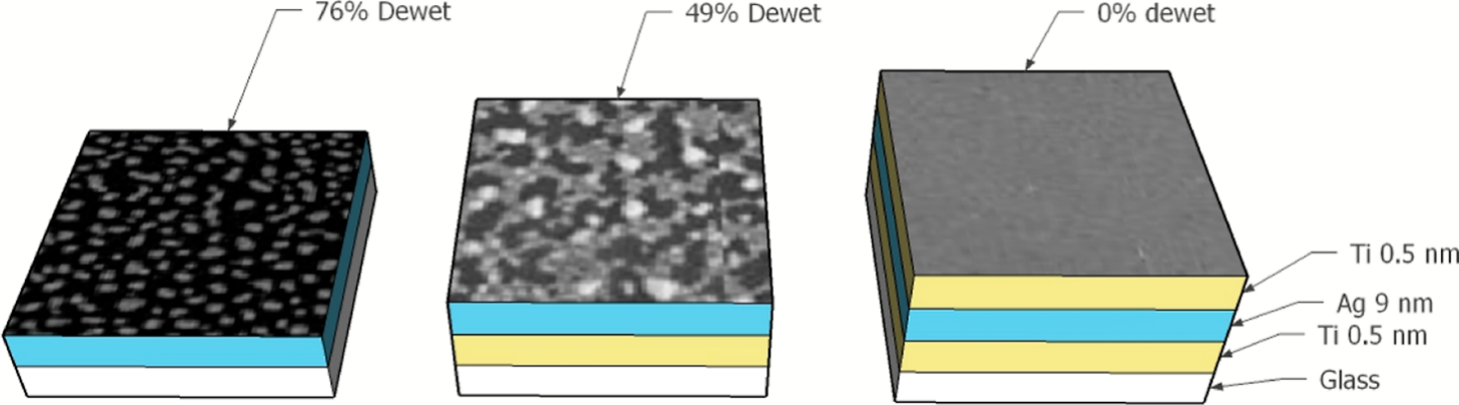

As technology development drives the thickness of thin film depositions down into the nano regime, understanding and controlling the dewetting of thin films has become essential for many applications. The dewetting of ultra-thin Ag (9 nm) films with Ti (0.5 nm) adhesion and capping layers on glass substrates was investigated in this work. Various thin film stacks were created using magnetron sputtering and were analyzed using scanning electron microscopy/energy dispersive X-rays, Vis/IR spectrometry, and four four-point probe resistivity measurements. Upon annealing for 5 h in air at 250 °C, the addition of a 0.5 nm thick Ti capping layer reduced the dewet area by an order of magnitude. This is reflected in film resistivity, which remained 2 orders of magnitude lower than uncapped variants. This Ti/Ag/Ti structure was then deployed in a typical low-emissivity window coating structure with additional antireflective layers of AZO, resulting in a superior performance upon annealing. These results demonstrate an easy, manufacturable process that improves the longevity of devices and products containing thin Ag films.

## Introduction

Silver films of thickness 10 nm or less are widely used in many applications such as thin film photovoltaics,^1,2^ flexible electronics,^3^ transparent conductors,^4^ antireflective coatings,^5^ transparent heaters,^3^ resistive temperature sensors,^6^ plasmonic devices,^7^ radiation detectors,^8^ optical and photonic devices,^9^ chemical sensing,^10^ and low emissivity (low-E) coatings.^1^ Low-E windows are commonly used to prevent heat loss or build up in buildings, and are a requirement under recent energy near zero building regulations.^11^ These windows block the majority of UV and IR light but still allow visible light to get through.^12^ Low-E coatings require films with low resistivity, high visible transmission, and high reflectivity in the infrared.^13^ Continuous silver films are commonly used for this application as they are conductive and transparent at the nano-scale with a low absorption coefficient.^14^ However, thin films of noble metals on oxide substrates are metastable and tend to dewet into patchy networks or discontinuous islands.^15^ This solid-state diffusion process is driven by energy reduction, and depends on interfacial energies, film thickness, and temperature.^16^ Dewetting involves a two-step process where hillocks are initially formed driven by compressive stress from thermal expansion mismatch.^17^ Holes are then formed by a rupture process. The hole size increases which leads to hole coalescence and dewetting.^18^

Dewetting can significantly degrade the optical and electronic properties of the film, and for some applications, it becomes a limiting factor in the lifetime or performance of devices. According to multiple window manufacturers, the lifetime of low-E windows is around 15 years, significantly shorter than the lifetime of buildings themselves. A technique to eliminate or retard dewetting could therefore significantly impact a broad range of applications, including low-E window lifetimes.

To retard dewetting, an adhesion layer of a few nanometers of Ti or Cr is normally applied between the desired metal film and the substrate. As these metals are more chemically reactive, they provide better adhesion through chemical bonding to the substrate. It has been shown that the optimal thickness of this Ti layer is c. 0.5 nm when used with 50 nm Au films.^19^ When Ti is used with a thickness of ≥5 nm, there can be negative consequences. It has been demonstrated that excess Ti is mobile in Au grain boundaries and can oxidize at the film surface leading to stress and film breakup.^20^ Ta and W are more stable, but all adhesion metals perform best when thickness is limited to c. 0.5 nm. Recently, capping layers were also found to help prevent dewetting, with sub-nanometre thickness (0.5–1 nm) of AlO_*x*_ proving most effective when used with 50 nm Au film stacks.^21^

In this work, we investigate the impact of c. 0.5 nm Ti adhesion and capping layers on the dewetting of 9 nm Ag films under thermal stress. We show that these layers can significantly reduce the degradation due to dewetting. These Ti/Ag/Ti stacks were then examined within a typical low emissivity coating stack containing aluminium doped zinc oxide (AZO) for possible use in low-E windows.

## Experimental Method

**Figure 1:**
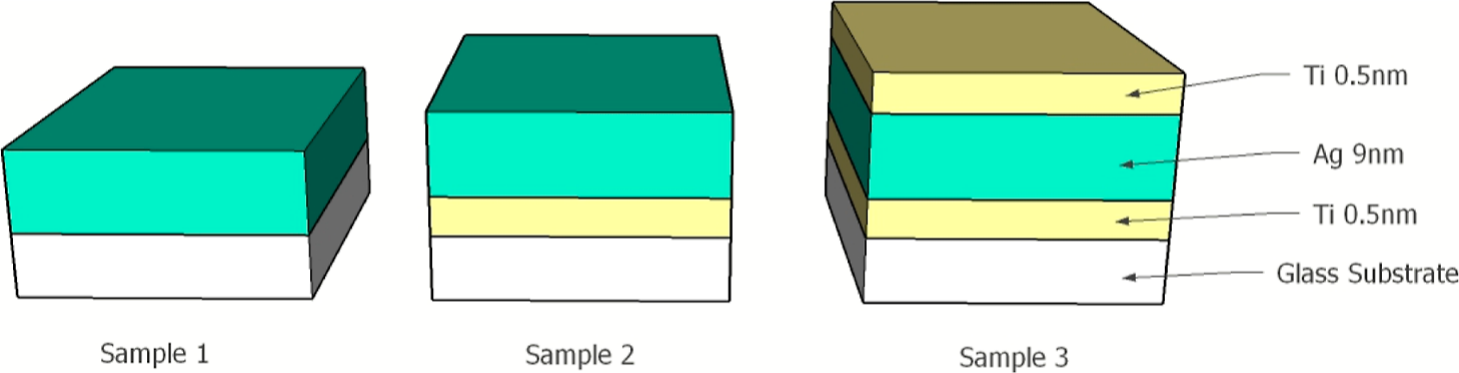
Experimental samples and their structure; sample 1—glass/Ag (9 nm); sample 2—glass/Ti (0.5 nm)/Ag (9 nm); and sample 3—glass/Ti (0.5 nm)/ Ag (9 nm)/ Ti (0.5 nm).

A magnetron sputtering system (Moorefield nanoPVD) with a base pressure of 1 × 10^–8^ mbar was used to deposit silver Ag (99.99% target purity) and Ti (99.995% target purity) layers onto glass slides at room temperature. The Ag target was deployed on a 2 in. DC gun at 12 W while Ti was deposited using a 2 in. RF gun at 75 W. An Ar partial pressure of c. 2 ×10^–3^ mbar was used during all depositions. In preparation, the thickness of calibration samples (c. 20 nm thick) using timed depositions was measured using low angle X-ray reflectometry (XRR- Phillips X’Pert system) and variable angle spectroscopic ellipsometry (J.A. Woolam Model α-SE). These thicknesses were then used to calculate the following deposition rates: Ag (0.11 nm/s) and Ti (0.06 nm/s). Glass slides were initially cleaned with acetone and then isopropyl alcohol in an ultrasonic bath and dried using a N_2_ gun prior to deposition. The Ag layer was deposited for 82 s for a target thickness of 9 nm, while the Ti was deposited for 9 s targeting 0.5 nm thickness. It is assumed that complete surface coverage is unlikely for these sub-nanometer films, and that the capping layer is completely oxidized. The structure of each sample type is shown in Figure 1.

Samples were annealed together on a hot plate in ambient conditions at a constant temperature of 250 °C for varying time intervals. This temperature was chosen so that measurable changes to the samples might take place over a period of minutes to hours rather than for a particular application. From previous work on thicker Au film systems,^19^ 250 °C was found to be a good choice. Film resistivity was measured initially and then after each time interval using a 4-point probe technique (Ossila). An integrating sphere spectrometer (PerkinElmer UV/vis Spectrometer Lambda 1050) was used to measure transmission spectra in the visible spectrum and a PerkinElmer FT-IR Spectrometer (Spectrum Two) to obtain the IR reflectivity and therefore the emissivity. Scanning electron microscopy (SEM) and energy dispersive X-rays (EDX) were used to analyze the morphology of the samples (Zeiss Ultra SEM with a Gemini column).

## Results and Discussion

**Figure 2:**
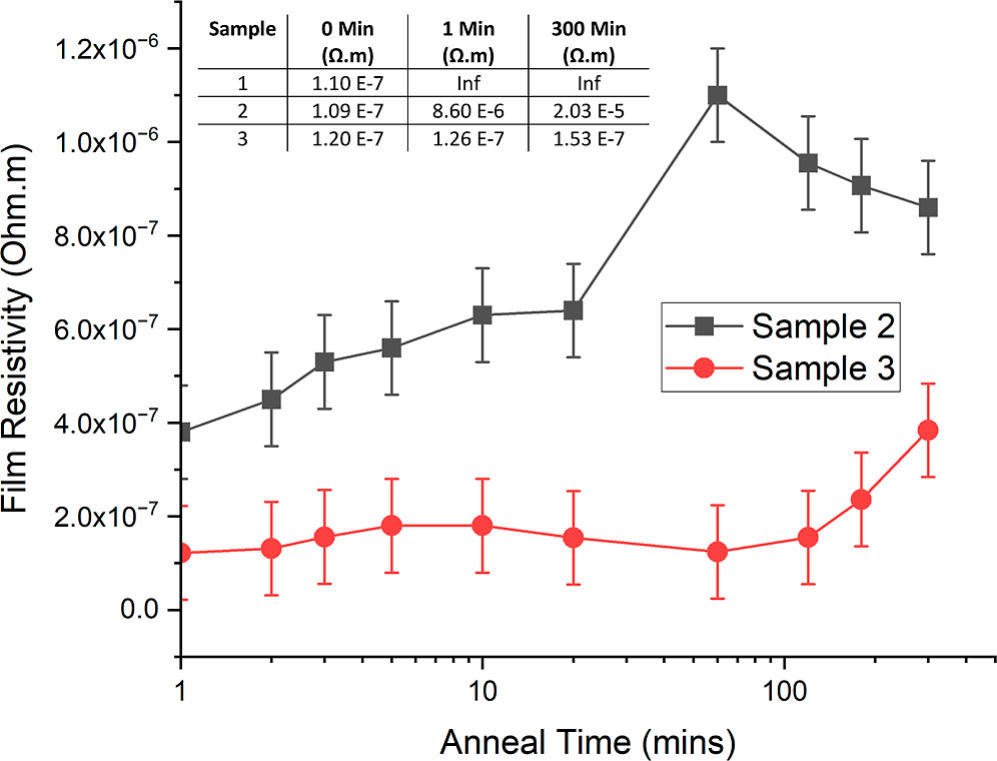
Samples 2 and 3 resistivity vs annealing time at 250 °C in air. Error bars indicate standard error. The inset table shows resistivity data at selected annealing times. Prior to annealing, all samples had similar resistivities.

Film resistivity measurements were carried out with three measurements per sample per annealing period. The initial resistivity of the samples was in the range 1.1–1.2 × 10^–7^ Ω m, which is comparable to literature values.^2^ After annealing for just 1 min at 250 °C, sample 1 returned infinite resistance suggesting substantial dewetting has already occurred. The resistivity of sample 2 increased by almost 2 orders of magnitude, while that of sample 3 increased by just 5%. Annealing continued for samples 2 and 3 only with regular resistivity measurements, and the results are shown in Figure 2. Sample 2 remained more resistive compared to sample 3, with a step change occurring somewhere between 20 and 50 min. As no further increase in resistivity was measured, dewetting had reached its full extent under these conditions. The last data point recorded was at 300 min by which time the resistivity of sample 2 became difficult to measure reliably while sample 3 remained relatively unperturbed.

**Figure 3:**
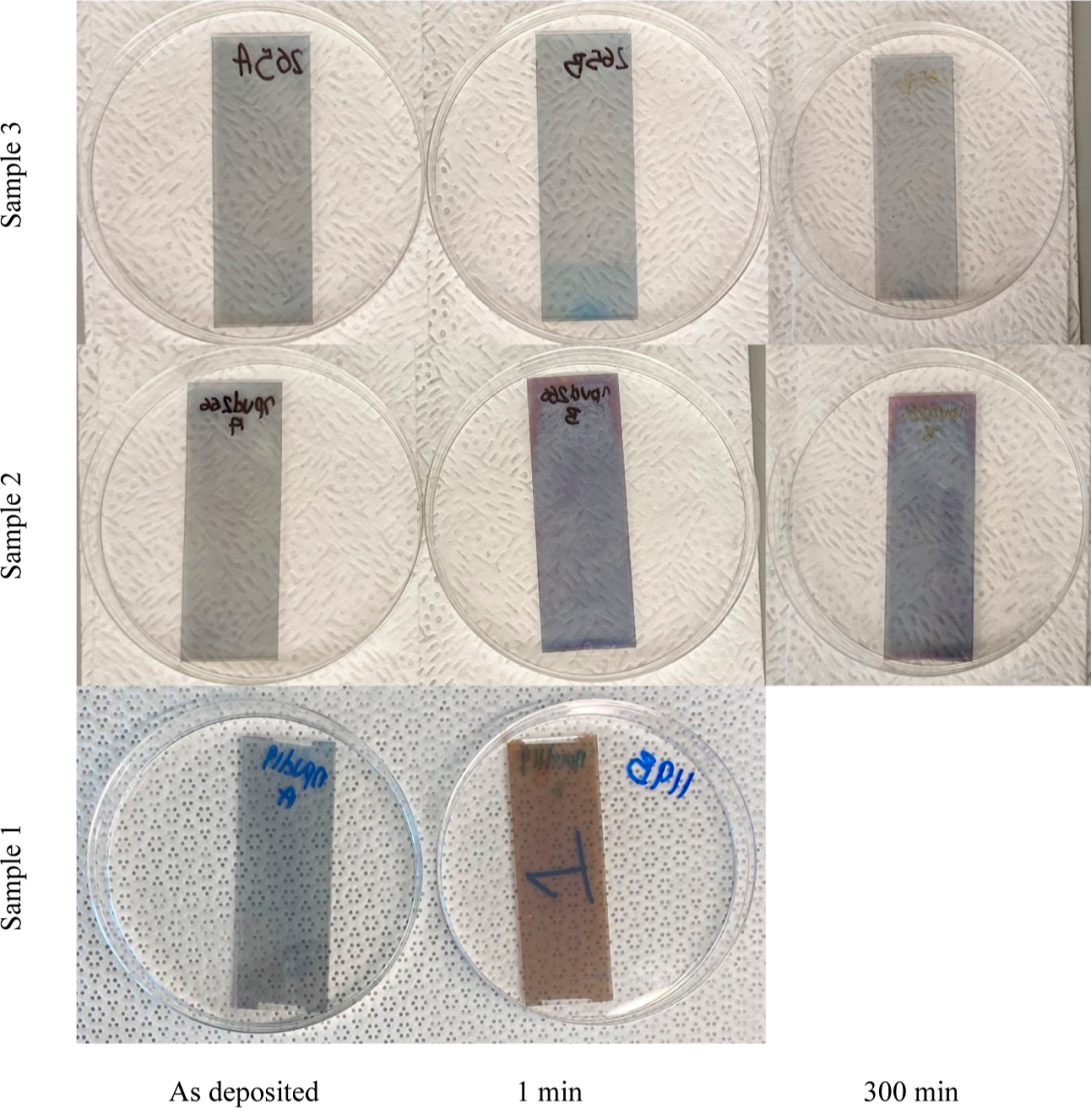
Samples 1, 2, and 3 as deposited and annealed for various periods on a hot plate in air at 250 °C.

After annealing for just 1 min at 250 °C, an obvious color change occurred for samples 1 and 2, suggesting some level of dewetting had already occurred (Figure 3). Sample 3 showed no noticeable difference. The reduction in optical transmittance and the color change, due to thermal annealing, is caused by the agglomeration of the thin film into hemispherical silver nanoparticles (NPs) that enhance both the intensity of Rayleigh scattering and the local surface plasmon resonance in the NPs.^22^ The latter, in particular, increases the absorption of the silver film. The radius, height, contact angle, and separation of these NPs is closely linked to both the anneal temperature and the initial silver film thickness^15^

**Figure 4:**
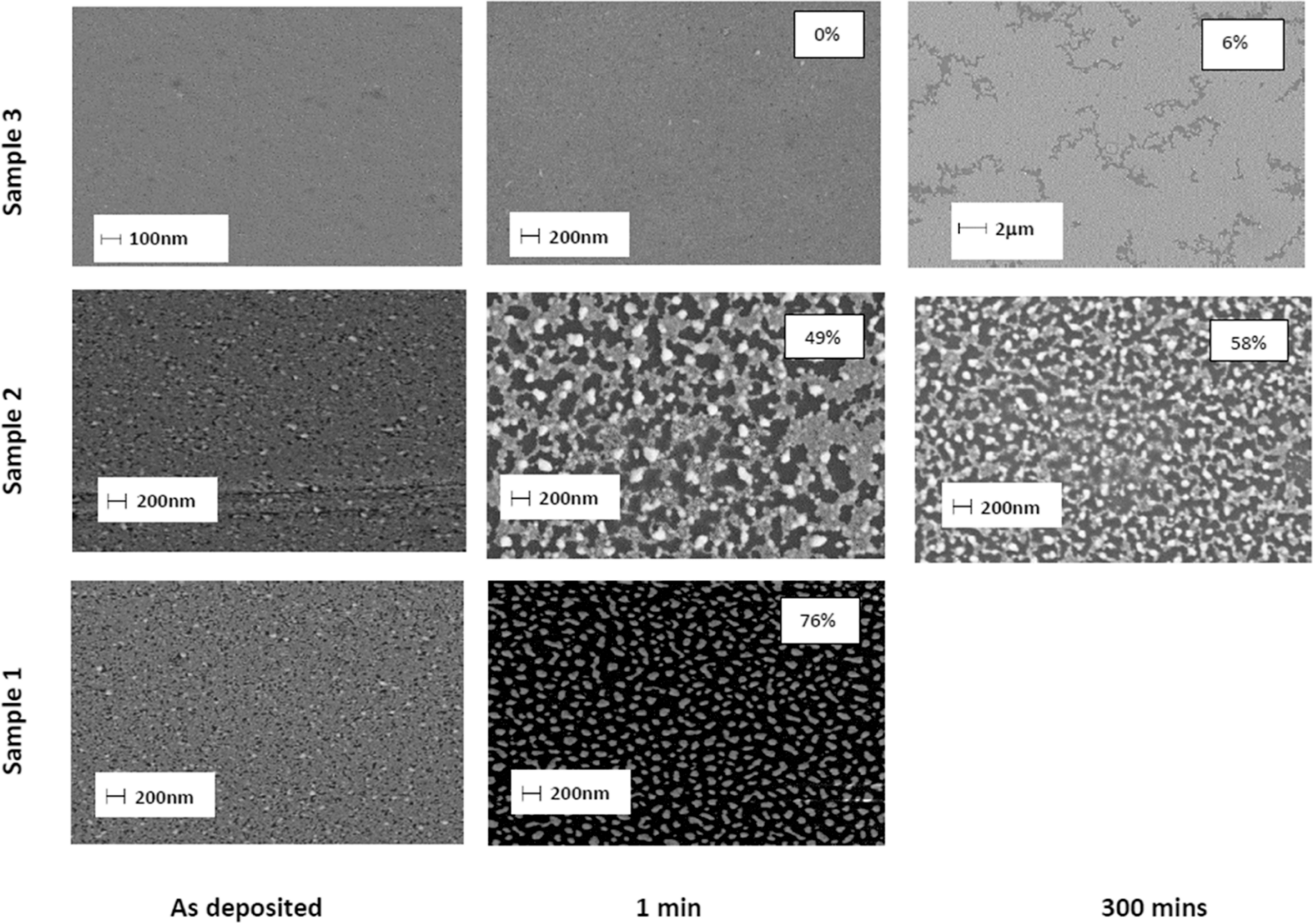
SEM images of samples 1, 2, and 3 annealed at 250 °C for various periods. The extent of dewetting for annealed samples is noted.

The extent of dewetting was evaluated using SEM, and the dewet area was quantified by image analysis (ImageJ software). The results are shown in Figure 4. These images, taken 5 days after sample fabrication, indicate that some dewetting has occurred even before annealing for samples 1 and 2, but none is apparent for sample 3. Upon annealing, sample 1 has been transformed into physically and electrically isolated Ag islands characteristic of substantial dewetting (76% bare substrate). The film has exceeded the percolation threshold which explains why resistivity is infinite after just 1 min at 250 °C.^23^ Sample 2 also underwent dewetting of up to 58% over the course of annealing, already 49% after just 1 min. Sample 3 on the other hand appears completely intact initially and dewets only slightly (6%) after annealing for 300 min. These results are consistent with measured changes in film resistivity.

The data shows that the deposition of a sub-nanometer Ti capping layer improves the dewetting resistance of silver thin films massively, by about an order of magnitude under these test conditions in terms of dewet area. An explanation for this effect has recently been published for 50 nm Au thin films,^21^ but the impact is much greater for these Ag films which are thinner and therefore more susceptible to dewetting. This protection against dewetting may have potential use in a wide range of applications. One such use case is low-E window coating stacks. Degradation of low-E windows over time is thought to be predominantly due to the solid state dewetting of the thin silver layer.^24^ A technique to eliminate or delay the onset of dewetting could therefore have a significant impact on the lifetime of the silver layer, making these windows more durable.

**Figure 5:**
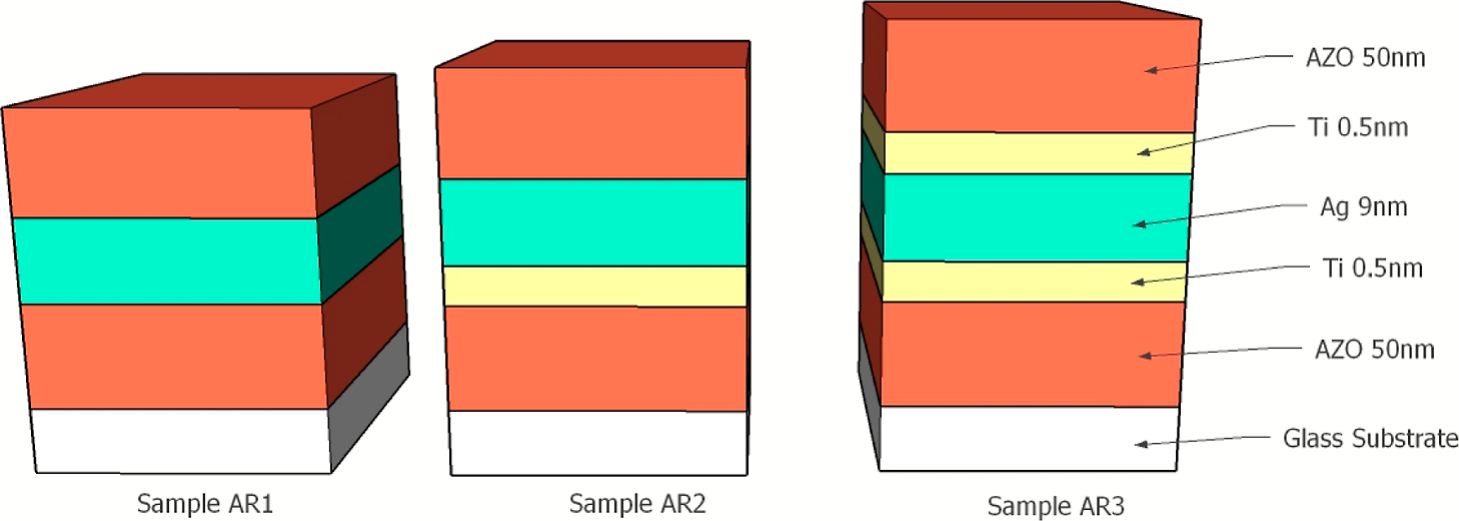
Antireflective stack structures consist of the stacks encapsulated in AZO.

**Figure 6:**
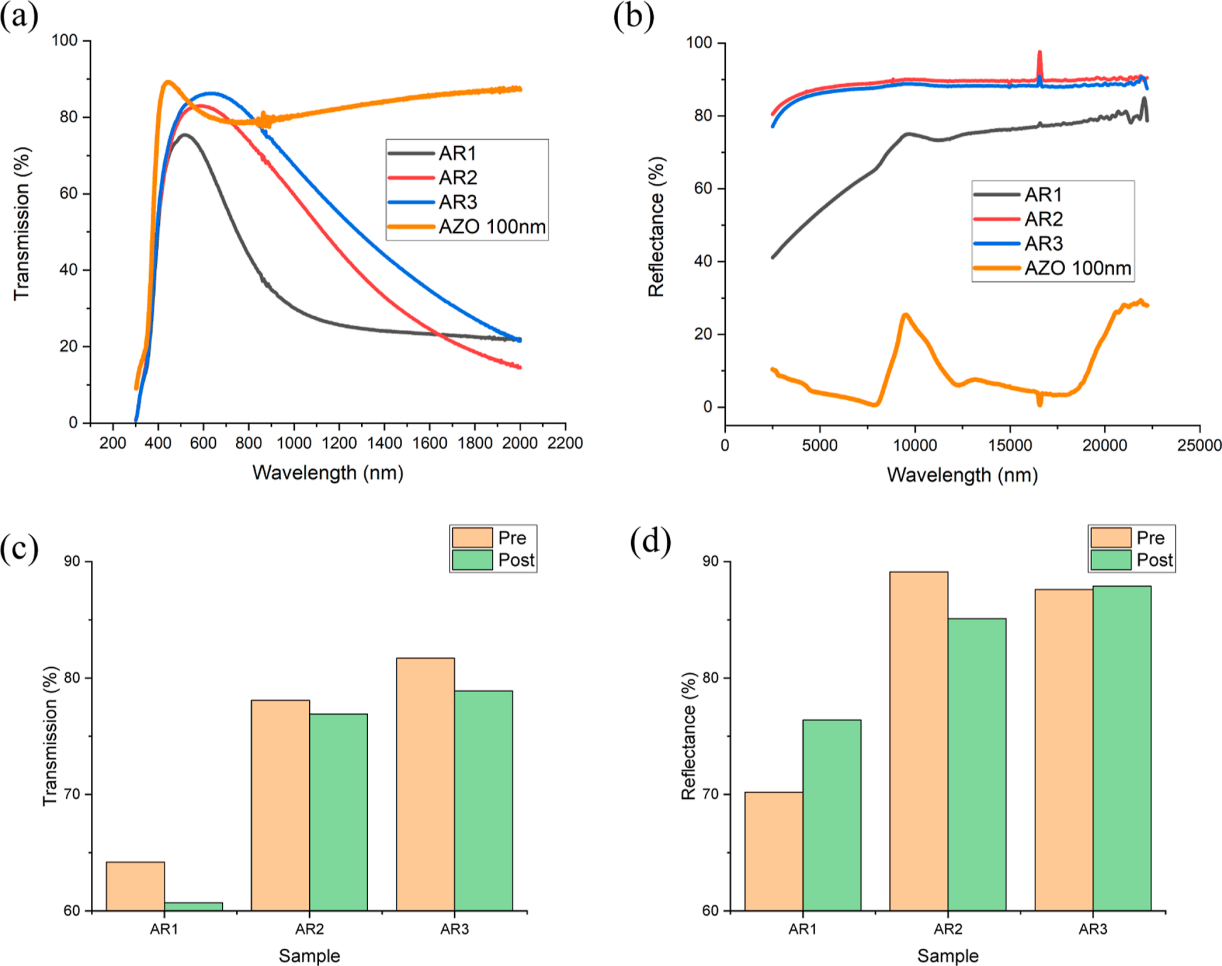
Visible transmission (a) and infrared reflection (b) spectra of the as-deposited samples and integrated visible transmission (c) and integrated infrared reflection (d) values pre/post annealing. High transmission in the visible and high reflectance in the infrared spectra are attractive characteristics for low-E coatings.

Sample stacks 1–3 were inserted into a typical low-e coating of dielectric/metal/dielectric stack to see if the above effect remains potent.^25^ AZO is a nontoxic, robust, transparent conductive oxide commonly used in thin film displays and photovoltaic applications due to its high visible transmittance, low electrical resistance, and low cost, compared to ITO, for example.^26^ The properties of AZO can be influenced by deposition parameters such as power, gas flow, substrate temperature, etc.^27^ The thickness of the AZO layers is important to enhance the visible transmission and antireflective properties. The ideal thicknesses of the individual layers were determined using a transfer matrix method approach which minimized total reflectivity of the stack structure [Figure S1]. Sample stacks 1–3 were prepared as before, with the addition of top and bottom 50 nm AZO layers (ZnO 98%/ Al_2_O_3_ 2%, 99.99% purity) deposited by RF sputtering. Figure 5 shows the sample stacks integrated with AZO. Spectra of the visible and infrared regions were taken of the as-deposited structures and are displayed in Figure 6a,b. For comparison, a control sample of AZO 100 nm on glass was also fabricated and measured. The stack samples were then annealed on a hot plate in air for 1 h at 250 °C, then a subsequent 1 h at 350 °C to assess their durability and remeasured. The integrated visible transmission and infrared reflection values pre- and post-annealing are shown in Figure 6c,d. Resistivity measurements are not useful due to the presence of the conducting AZO layers.

All samples remained relatively stable after annealing. When compared to the samples that were not encapsulated in AZO, the changes are much smaller, particularly in infrared reflectance. This suggests that AZO encapsulation also contributes substantially to dewetting resistance.

Prior to annealing, it is seen that the sample with Ti capping and adhesion layers has the highest visible transmission compared to samples with only the adhesion layer or with no capping or adhesion layer. By comparison, the AZO 100 nm control sample remains transmissive out to the measurement limit of 2000 nm.^28^ While the infrared reflectance is best with the adhesion layer only , the reflectivity for AR3 is still a very high value. The best performing low-e coating is the sample with both adhesion and capping layer of 0.5 nm Ti. This is likely due to the Ag layer being more continuous with the capping and adhesion layer, thereby improving the overall transmission. The AZO 100 nm control sample has much lower reflectance across the IR range.^29^

**Figure 7:**
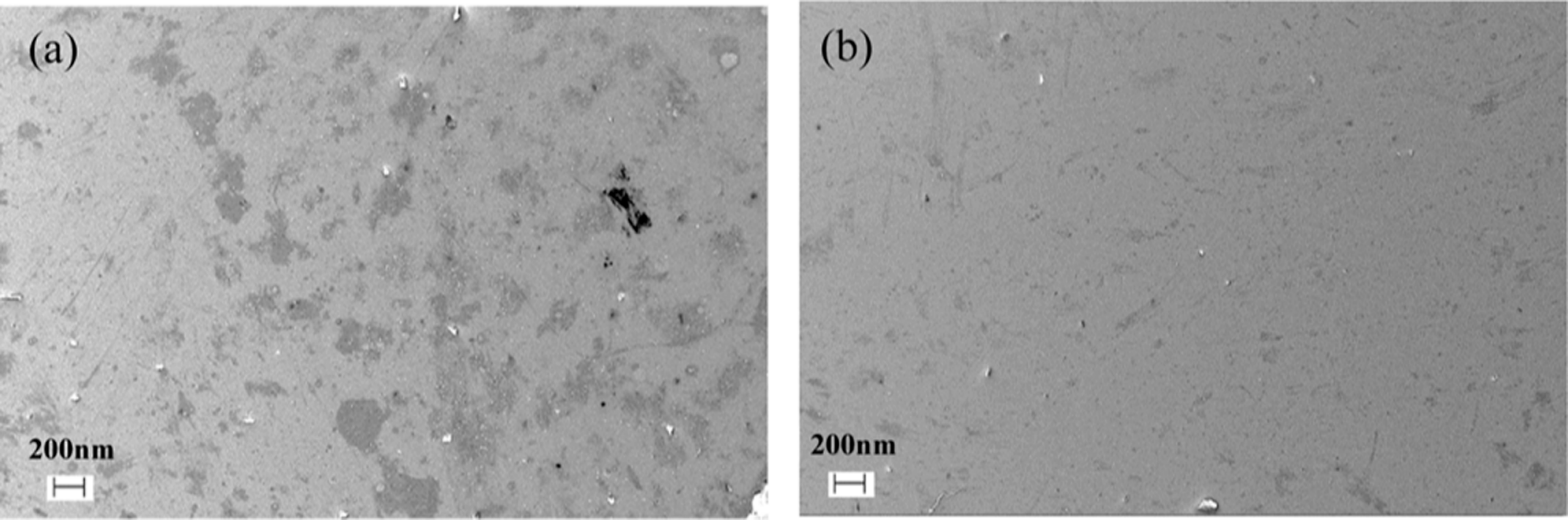
SEM images of (a) sample AR2 and (b) sample AR3 after for 1 h 400 °C on a hot plate in air. Darker areas are due to dewetting of the Ag layer.

To clarify this, sample AR2 was compared to sample AR3 to establish the capping layer contribution to dewetting in the AZO stack. To force dewetting, both samples were annealed for 1 h at 400 °C. SEM was performed using a Zeiss Ultra operating at 5 kV to analyze the morphology of the samples (Figure 7), and elemental analysis was acquired using an Oxford instruments EDX system. Sample AR3 showed little contrast difference across the sample, with the 3 keV Ag EDX peak present. There was a clear contrast difference across the AR2 sample. EDX shows that lighter gray regions were silver rich, while the darker gray regions were silver poor (Figure S2). This indicates that some dewetting has taken place, although not to the same extent, as shown in Figure 4 above. This suggests that the 0.5 nm Ti capping layer remains beneficial in increasing visible transmission and preventing Ag dewetting even when encapsulated by AZO, but not to the same extent as non-AZO encapsulated variants.

## Conclusions

Capping Ti (0.5 nm)/ Ag (9 nm) thin film stacks on glass substrates with a Ti 0.5 nm layer has been shown to reduce dewetting by a factor of 10x in terms of dewet surface area, while preventing a 100× increase in film resistivity compared to uncapped variants, under thermal stress. Inserting these stacks in an optimized low-emissivity AZO sandwich structure resulted in a high visible transmission of 81.7% and infrared reflection of 87.6%. SEM/EDX analysis indicates that adding Ti capping adds to dewetting prevention even when inserted into AZO layers compared to a sample with only an adhesion layer. Other use cases where encapsulation is not required may benefit to a greater degree. This method provides an easy fabrication route toward extending and improving the lifetime and performance of a range of thin-film applications and devices.

## Supporting Information Available

Description of the methods used to calculate optimal film thickness for use in antireflective coating samples and SEM images and EDX analysis of dewet and nondewet areas of an antireflective coating stack (Ag dewetting) (PDF)

## Abbreviations

SEM: scanning electron microscopy
EDX: energy dispersive X-rays
