# Supplementary material for: Extreme Dewetting Resistance and Improved Visible Transmission of Ag Layers Using Sub-Nanometer Ti Capping Layers
Source: ACS Omega. 2024 Feb 15;9(8):9714–9. doi: 10.1021/acsomega.3c09774 (PMC10905571; doi:10.1021/acsomega.3c09774)
Supplement: Supplementary file 1 — ao3c09774_si_001.pdf [file ao3c09774_si_001.pdf]

Supplemental Information

Extreme De-Wetting Resistance and Improved Visible Transmission of Ag Layers using Sub-Nanometer Ti Capping Layers

Amy L. Lynch[1], Christopher P. Murray[2*], Evan Roy[2], Clive Downing[2] and David McCloskey[2]

1 Technical University of Dublin, Grangegorman, Dublin D07 H6K8, Ireland

2 School of Physics, Trinity College, Dublin D02 PN40, Ireland

*Corresponding author: murrayc2@tcd.ie

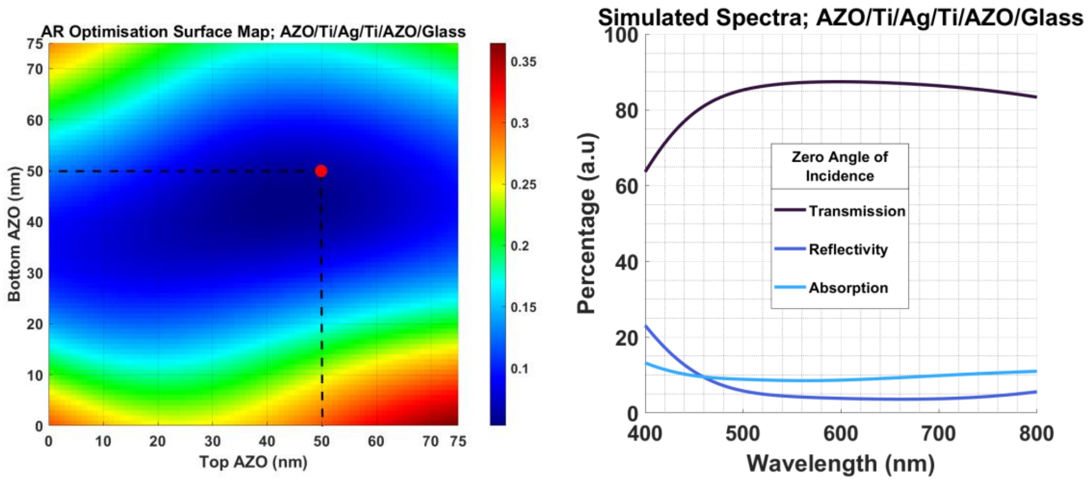

Figure S1: The results of the AR coating TMM optimisation program written in Matlab code. The optimisation was performed iteratively for zero incident angle using refractive index data obtained from a J.A Wollam Alpha SE ellipsometer tool. The 1 mm thick glass was modelled as an incoherent layer to account for substrate back reflections. The simulated zero incident angle spectrum is based on a TMM simulation of a 50 nm AZO/0.5 nm Ti/9 nm Ag/0.5 nm Ti/50 nm AZO stack structure.

These simulated results above in Figure S1 are derived from code that was written up in Matlab due to the languages' efficiency in processing large matrix calculations. The transfer matrix method (TMM) is an efficient method for calculating optical reflectivity, transmission, and absorption of planar stack structures. The TMM is based on the Fresnel equations, wave optics interference, and incorporates Maxwell relations for layer boundary conditions and field propagation in different media[1,2,3,4]. From this approach an iterative optimisation program was written that searched through many layer permutations to find the stack structure that minimised reflectivity averaged across the 400 nm – 800 nm visible spectrum. The large search was narrowed down by considering certain physical constraints that are relevant to thin film anti-reflection (AR) coatings, such as satisfying the quarter-wave film condition needed for destructive interference of the light incoming and out-going from the stack structure. Traditionally a single low index layer such as MgF_2 at the air/glass interface

is used to satisfy the quarter-wave film condition. This condition can still be met with the higher refractive index materials like AZO (n > 2), with which average reflectivity across the visible can be reduced to near 5%. The addition of a lower index layer above the top AZO layer such as Al_2O_3, or the MgF_2, could improve performance further by protecting the conducting layers underneath from the environment and broadening the reduction in reflectivity more evenly across the visible spectrum. However, in so doing the stack structure complexity would increase. Other meta surface like AR methods can also be incorporated into the design with subwavelength texturing of the surface (e.g. nanoscopic texturing and porous dielectric layers). This effectively creates a gradient index film, with different properties than bulk, that provides better impedance matching between the air and the stack structure underneath[5,6].

Figure S2 shows SEM images and EDX spectra of selected regions of a sample AR2 annealed for 1 hour at 400°C. Contrast differences are indicative of dewetting of the Ag layer. Spectrum 1 is taken from a dark region and indicates low/zero Ag presence. Spectrum 2 is from a lighter region where Ag is clearly detected. Darker regions are thus confirmed to be areas where Ag has dewet.

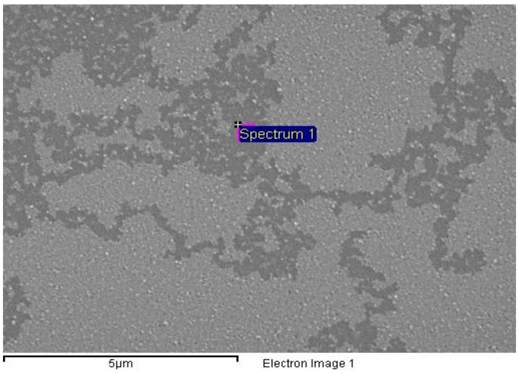

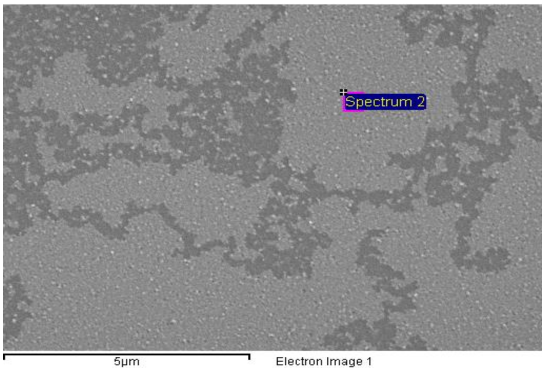

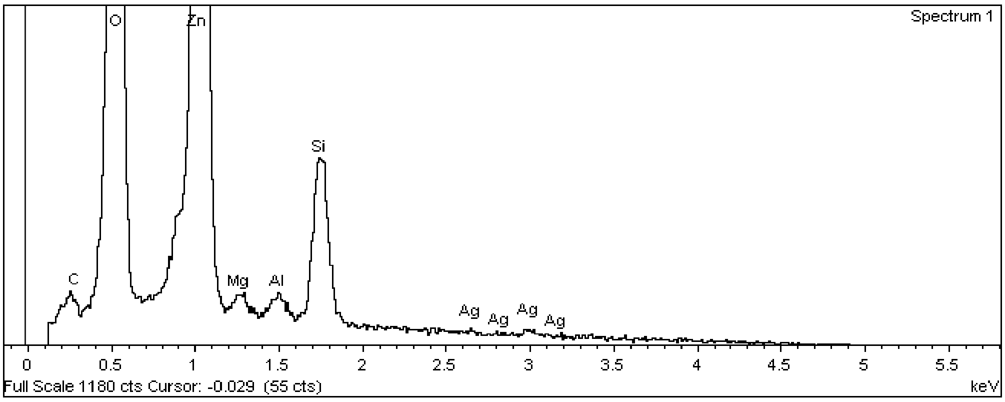

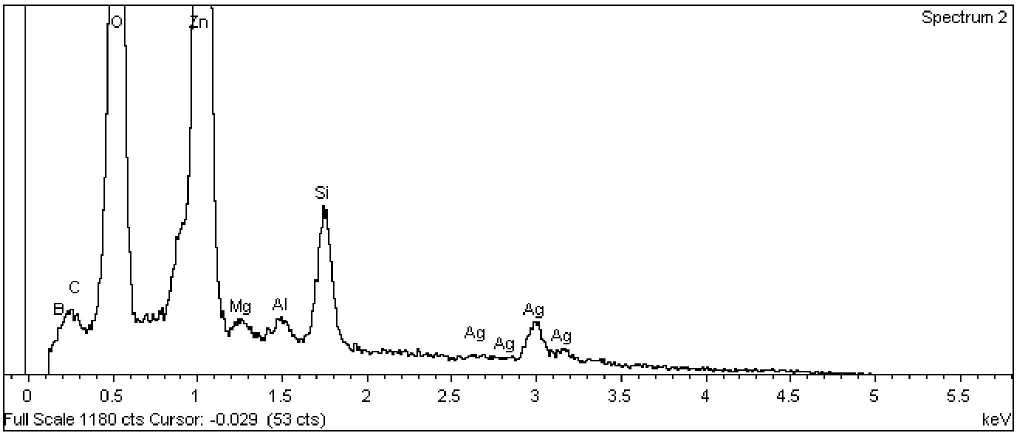

Figure S2: EDX results showing the difference between light and dark regions of Figure 9 SEM images of sample AR2 after 400°C annealing on a hot plate for one hour. An accelerating voltage of 5kV is used. Darker regions are shown to be low in Ag due to dewetting.

References

[1] Katsidis, C. C., & Siapkas, D. I. (2002). General transfer-matrix method for optical multilayer systems with coherent, partially coherent, and incoherent interference. *Applied Optics*, *41*(19), 3978–3987.

[2] Santbergen, R., Smets, A. H. M., & Zeman, M. (2013). *Optical model for multilayer structures with coherent , partly coherent and incoherent layers*. *21*(March), 262–267.

[3] Kenneth J. Pascoe. (2001). *Reflectivity and Transmissivity through Layered, Lossy Media A User-Friendly Approach*. Graduate School of Engineering and Management Air Force Institute of technology Wright-Patterson Air Force base Ohio.

[4] Byrnes, S. J. (2016). *Multilayer optical calculations*. http://arxiv.org/abs/1603.02720

[5] Rahman, A., Ashraf, A., Xin, H., Tong, X., Sutter, P., Eisaman, M. D., & Black, C. T. (2015). Sub-50-nm self-assembled nanotextures for enhanced broadband antireflection in silicon solar cells. *Nature Communications*, *6*. https://doi.org/10.1038/ncomms6963

[6] Ruud, C. J., Cleri, A., Maria, J. P., & Giebink, N. C. (2022). Ultralow Index SiO2Antireflection Coatings Produced via Magnetron Sputtering. *Nano Letters*, *22*(18), 7358–7362. https://doi.org/10.1021/acs.nanolett.2c01945
